# Phytochemical Screening Using LC-MS to Study Antioxidant and Toxicity Potential of Methanolic Extracts of *Atraphaxis pyrifolia* Bunge

**DOI:** 10.3390/molecules29184478

**Published:** 2024-09-20

**Authors:** Alima Abilkassymova, Jennyfer A. Aldana-Mejía, Kumar Katragunta, Raushan Kozykeyeva, Ardak Omarbekova, Bharathi Avula, Aknur Turgumbayeva, Ubaidilla M. Datkhayev, Ikhlas A. Khan, Samir A. Ross

**Affiliations:** 1 Higher School of Medicine, Al-Farabi Kazakh National University, Almaty 050040, Kazakhstan; abilkasymova_a@mail.ru (A.A.); turgumbayeva.aknur@med-kaznu.com (A.T.); 2 School of Pharmacy, Asfendiyarov Kazakh National Medical University, Almaty 050012, Kazakhstan; ardashka.0892@mail.ru (A.O.); u.datkhayev@gmail.com (U.M.D.); 3 National Center for Natural Products Research, School of Pharmacy, The University of Mississippi, Oxford, MS 38677, USA; jaaldana@olemiss.edu (J.A.A.-M.); kkatragu@olemiss.edu (K.K.); nar_rau@mail.ru (R.K.); bavula@olemiss.edu (B.A.); khan@olemiss.edu (I.A.K.); 4 Faculty of Pharmacy, South Kazakhstan Medical Academy, Shymkent 160019, Kazakhstan; 5 Department of Biomolecular Sciences, Division of Pharmacognosy, School of Pharmacy, University of Mississippi, Oxford, MS 38677, USA

**Keywords:** antioxidant, cytotoxicity, glycosylated flavonoids, *Atraphaxis* species, therapeutic applications

## Abstract

*Atraphaxis pyrifolia*, a native medicinal plant of Central Asia, has a long history of traditional medicinal use; however, scientific research on its phytochemical and biological properties remains scarce. This paper aims to elucidate its chemical profile and assess its pharmacological potential through a comprehensive investigation of the phytochemical composition of stems and leaves using Liquid Chromatography-Mass Spectrometry (LC-MS), in conjunction with the assessment of its antioxidant (DPPH and ABTS) and cytotoxicity test on *Artemia salina*. Predominantly, glycosylated flavonoids were detected in stems and leaves extracts, notably including 8-Acetoxy-3′,4′,5,5′-tetrahydroxy-7-methoxy-3-α-L-rhamno-pyranosyloxyflavone, pyrifolin, and dehydroxypyrifolin. While the latter compound is exclusive to *A. pyrifolia*, the former compounds serve as shared chemical markers with other *Atraphaxis* species. The methanolic extracts of *A. pyrifolia* leaves exhibited significant antioxidant capacity without toxicity against *Artemia salina*. This study contributes to current research through providing valuable insights into the chemical diversity and potential medicinal properties of this plant species.

## 1. Introduction

The genus *Atraphaxis* L., belonging to the Polygonaceae family, encompasses 47 species [1] distributed predominantly across arid and semi-arid regions, particularly in Asia and Eastern Europe [2]. Among these species, *Atraphaxis pyrifolia* Bunge holds a notable position due to its historical utilization in traditional medicine and its potential therapeutic significance [3]. According to traditional Uzbekistan medicinal practices, drinking an infusion of *A. pyrifolia* leaves can potentially support cardiovascular health, improve circulation, and alleviate symptoms associated with headaches, insomnia, and tinnitus [4]. It may also enhance overall bodily tone [4]. This species has been utilized in the traditional medicine of Afghanistan, Kazakhstan, China, and other parts of Central Asia for the treatment of respiratory and skin conditions [3]. Despite its longstanding traditional use, the molecular basis underlying its medicinal properties remains largely unexplored.

Previous studies on the phytochemistry of the genus *Atraphaxis* have demonstrated the presence of a wide range of bioactive substances, including tannins, phenolic compounds, anthraquinones, phenylpropanoids, and flavonoids [3,5]. Phytochemical screenings of *A. pyrifolia* revealed a major presence of flavonoid glycosides [6,7,8,9]. And recent GC-MS studies of the hexane extract from *A. pyrifolia* leaves revealed the presence of γ-sitosterol and lup-20(29)-en-3-one, as well as the alkane nonacosane as the main nonpolar compounds [10]. In addition, our research group isolated and identified, for the first time, two flavonoid glycoside from the methanolic leaf extract of this species [10].

Phytochemical screening allows for the identification of bioactive compounds within plant sources, which may have potential pharmaceutical applications. *A. pyrifolia* studies have been focused on classical phytochemical techniques such as Vacuum Liquid Chromatography, Sephadex LH-20, and HPTLC [10]; however, these methods can have some limitations in terms of sensitivity, time, and solvent consumption [11]. Liquid Chromatography-Mass Spectrometry (LC-MS) has revolutionized phytochemical analysis, enabling a faster identification and quantification of diverse phytoconstituents with remarkable sensitivity and precision [11].

This study investigates the phytochemical and biological properties of *A. pyrifolia*. It focuses on the methanolic extracts of its stem and leaves, using LC-MS analysis, and conducts antioxidant and toxicity assessments. The comprehensive characterization of secondary metabolites present in *A. pyrifolia* not only contributes to the phytochemical knowledge base but also highlights the significance of this species as a reservoir of bioactive compounds with promising medicinal attributes.

## 2. Results

### 2.1. Phytochemical Screening

**Figure 1:**
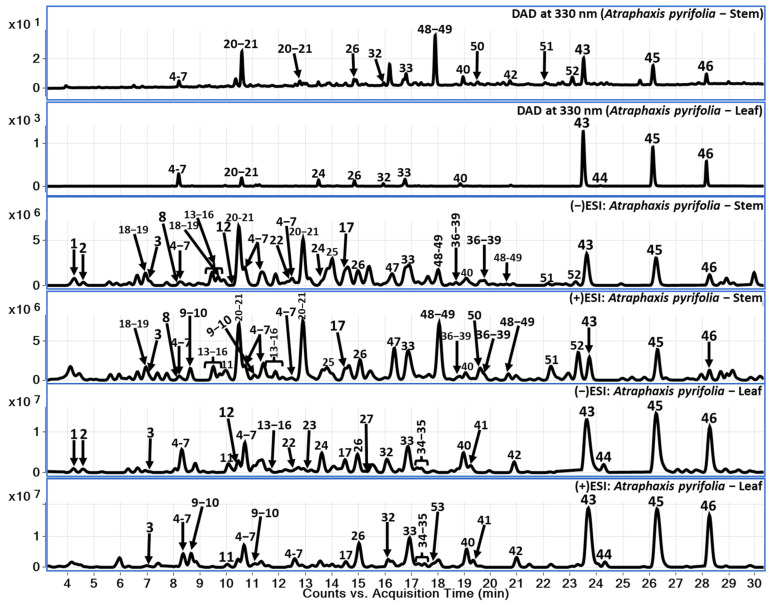
LC-DAD at 330 nm and TCC (positive and negative) for *A. pyrifolia* leaf and stem methanolic extracts.

Chemical constituents of *A. pyrifolia* aerial parts (stem and leaves) were analyzed using reverse phase liquid chromatography in gradient elution mode followed by the identification of secondary metabolites using QToF-MS analysis in hyphenation. Here, we have used electrospray ionization-high resolution mass spectrometry (ESI-QToF-MS). The LC-QToF-MS method facilitates the tentative identification of chemical constituents including phenolic compounds (**1–12**), flavonoids (**13–46**), catecholamines (**47–52**), and monoterpenoid lactone compound (**53**). Further, within the flavonoid class of compounds, procyanidins (**13–17**), catechins (**18–22**, **25**, **28–31**, and **36–39**), dihydroflavonol (**46**), and flavonols (**23**, **24**, **26**, **27**, **32–35**, and **40–45**) were identified. The identification of compounds using the LC-QToF-MS method of analysis in this study was divided into two levels: identification through comparison with reference standards and tentative identification using the mass spectral data and previously reported literature [12]. Specifically, compounds were identified based on comparison of MS data and retention time with reference standards (Compounds **42** and **45–46**), while others were tentatively identified and annotated based on mass spectral data reported in the literature (Compounds **1–41**, **43**, and **47–53**). Major compounds observed in leaf part are flavonols whereas these compounds (major flavonols) were observed in low amounts in the stem sample. Data analyses were performed in both positive and negative modes to ensure the identification of the compounds and to confirm the better ionization of the secondary metabolites from *A. pyrifolia* stem and leaf samples. Tentatively identified compounds are listed in Table 1, including their retention time, molecular formula, and extract mass in both positive and negative modes wherever it was applicable, along with their observed fragment ions. A total of 53 compounds were tentatively identified based on their exact mass, fragment ions, and retention patterns. Considering the major flavonoids in both stem and leaf samples, respective LC-DAD chromatograms were presented at 330 nm (Figure 1) along with total current chromatograms (TCC) in both positive and negative ionization modes to provide a comparison.

#### 2.1.1. Phenolic Compounds (**1–12**)

A total of twelve phenolic compounds were identified based on the characteristic fragments observed, respective to the precursor ions observed. Chlorogenic acid isomers (compounds **4–7**) are the major compounds observed in both stem and leaf parts of *A. pyrifolia* methanolic extracts. The identified phenolic compounds contain gallic acid derivatives, coumaric acid derivatives, and vanillic acid derivatives, along with the quinic acid derivatives. Gallic acid hexoside (**1**) showed a *m*/*z* 331.0673 [M-H]^−^ precursor ion along with characteristic fragment ions of *m*/*z* 169.0144 [M-H-Glc]^−^ and 151.0035 [M-H-Glc-H_2_O]^−^. Similarly, other phenolic compounds (**2–12**) were tentatively identified and listed along with the observed molecular features along with their chromatographic retention times in Table 1 [13,14].

#### 2.1.2. Flavonoids (**13–46**)

**Figure 2:**
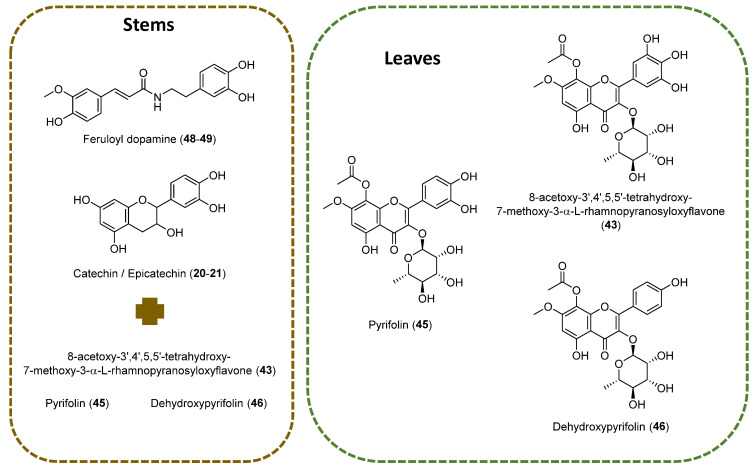
Main chemical structures identified on stems and leaves of *A. pyrifolia* methanolic extracts using LC-MS.

*A. pyrifolia* leaf extract showed the predominance of flavonoid compounds in comparison with the stem part (Figure 2). Various flavonoids class of compounds such as procyanidins (**13–17**), catechins (**18–22**, **25**, **28–31**, **36–39**), flavonols (**23**, **24**, **26**, **27**, **32–35**, **40–45**), and dihydroflavonol (**46**) were identified. Compound **43** shows *m*/*z* 535.1105 precursor ion with substantial fragment ions of *m*/*z* 493.0995 [M-H-C_2_H_2_O]^−^, indicating terminal ester bond breakage; this is followed by *m*/*z* 388.0442, which indicates a loss of rhamnose moiety and a further loss of methyl group. Based on the observation of fragment ions, the literature of previously reported compounds from *A. pyrifolia* and observed the molecular features of the compound **43** tentatively identified as 3,3′,4′,5,5′,7,8-Heptahydroxyflavone, alongisde 7-Me ether, 8-Ac, 3-*O*-α-L-rhamnopyranoside. Compounds **45** and **46** were identified as pyrifolin and dehydroxypyrifolin, respectively, through comparing with the reference standards isolated from our previously reported study [10]. The identified fragment ions and the chromatographic retention time information were listed in Table 1 for the identified compounds. These major compounds belong to the flavonols class.

Further, a few minor flavonol compounds were identified along with polyhydroxy flavones which contains quercetin, kaempferol, myricetin, and fisetinidol derivatives [15]. As mentioned earlier, the second subclass of compounds belongs to bioflavonoids which are procyanidin derivatives that show a precursor ions at *m*/*z* 577.1356 [M-H]^−^ and 729.1463 [M-H]^−^, which belong to galloyl procyandin derivative at 14.47 min.

#### 2.1.3. Catecholamines (**47–52**)

A total of six catecholamine compounds were identified, and the majority was observed in the stem extract in comparison with the leaf extract. The *m*/*z* 330.1333 [M + H]^+^ precursor ion was identified at 18.0 min and 20.66 min. Based on the chemical formula and characteristics, the fragment ion *m*/*z* 177.0543 was observed from the precursor ion of *m*/*z* 330.1333, indicating the presence of feruloyldopamine compounds (compound **48–49**). Similarly, other catecholamine compounds such as a coumaroyl derivative of dopamine, feruloyl derivatives of tyramine, and a feruloyl derivative of noradrenaline were tentatively identified based on their mass spectra along with fragment ions [5,16]. In addition to these catecholamine compounds, one monoterpenoid lactone compound was identified with *m*/*z* 197.1172 as a precursor ion and confirmed as loliolide (**53**).

### 2.2. Antioxidant Capacity

The antioxidant capacity results of the extracts were determined using two different methods: DPPH and ABTS (Table 2). The results indicated that the *A. pyrifolia* leaves extract showed more activity than the stem extract in both assays.

### 2.3. Toxicity Assay

The results of the cytotoxic activity studies are presented in Table 3. The methanol extract of the leaves of the plant *A. pyrifolia* Bunge did not exhibit any cytotoxic effects. In contrast, the reference drug Actinomycin D demonstrated significant cytotoxicity towards the marine crustaceans *A. salina* across all tested concentrations, resulting in larval mortality rates ranging from 63% to 96%.

## 3. Discussion

The findings of the phytochemical and biological screenings hold significant importance, particularly due to the limited understanding surrounding the genus under investigation. While the existing literature has briefly addressed the isolation and characterization of compounds from the aerial parts of *A. pyrifolia* [6,7,8,9,10,17], our study represents a comprehensive effort towards elucidating the chemical profile of this species, and preliminary evidence supporting its potential pharmaceutical characteristics.

The results revealed that flavonoids, particularly gallocatechin/epigallocatechin (Compounds **20–21**) and feruloyldopamine (**48–49**), were predominant in the stems extract. These compounds have previously been identified in aerial parts of *Atraphaxis frutescens* [18], highlighting a broader distribution of these phytochemicals within the genus. On the LC-MS analysis of the leaves, three main compounds were identified: 8-Acetoxy-3′,4′,5,5′-tetrahydroxy-7-methoxy-3-α-L-rhamnopyranosyloxyflavone (**43**), pyrifolin (**45**), and dehydroxypyrifolin (**46**). Compound **43** has been previously isolated from aerial parts of *A. frutescens* [18]; however, this is the first report of its presence in *A. pyrifolia*. The detection of this compound as well as pyrifolin in both *A. frutescens* [18] and *A. pyrifolia* [8,10] confirms the presence of shared chemical profiles among closely related species. The aglycones of this class of compounds have also been identified in species of the genus *Atraphaxis*. For example, 8-*O*-Acetyl-7-*O*-methylgossypetin has been isolated from *A. laetevirens* [19].

The identification of novel chemical constituents, such as dehydroxypyrifolin (**45**) in *A. pyrifolia* as reported by us previously [10], expands our understanding of its phytochemical profile. Additionally, a minor related compound, 3,4′5,7,8-pentahydroxyflavone, 7-Me ether, 3-*O*-α-L-rhamnopyranoside (**42**), previously isolated from *A. pyrifolia* [10], was corroborated using LC-MS analysis at 20.96 min, with a m/z 461.1097 precursor ion.

Additionally, the presence of several flavonoids and phenolic compounds on the sample underscores both the chemical consistency and variation within the genus. Methyl gallate (**12**) and gallic acid (**2**) were documented in *A. spinosa* [5]; fisetinidol 3′-*O*-*β*-D-glucopyranoside (**22**) was isolated from *A. frutescens* [18]; quercetin 3-*O*-*β*-D-glucuronide-6″-methyl ester (**23**) is present in *A. spinosa* [5]; 7-methylgossypetin 8-*β*-D-glucopyranoside (**26**) was reported in *A. pyrifolia* [7]; 7-*O*-methylluteolin 4′-*O*-*β*-D-glucofuranosyl-(1→6)-D-glucopyranoside (**32**) is in *A. spinosa* [6]; and myricetin 3-*O*-*β*-D-glucopyranoside (**27**) was isolated from the aerial parts of *A. virgata* [20].

The consistency of some cited flavonoids across various species within the genus suggests a degree of chemical homogeneity, indicating their potential as chemical markers, while also highlighting potential variations that may reflect species-specific adaptations or ecological influences. However, additional data, comprehensive analyses, and comparisons among multiple species are required to confirm this. Despite this, the findings underscore the potential of chemical characterization as a valuable tool in chemotaxonomy, providing important insights that can be applied in future studies to explore species relationships.

This chemical diversity also holds relevance in the context of the pharmacological potential of these species. Enriched fractions containing gallocatechin gallate (GCG) and epigallocatechin gallate (EGCG) have exhibited significant antiviral activity against various viruses, including influenza A virus, feline calicivirus, murine norovirus, and SARS-CoV-2 [21], underscoring their promise as antiviral agents. Our analysis has confirmed their presence in both stems and leaves of *A. pyrifolia*.

Furthermore, gallic acid has been acknowledged for its potential as an anticancer agent [22,23] as well as for its anti-inflammatory, antioxidant properties, and its capacity to inhibit osteoclastogenesis, a process implicated in pathological conditions such as osteoporosis and autoimmune arthritis, among others [24]. Conversely, methyl gallate (**12**), present in our sample, has been associated with anti-tumor, anti-inflammatory, antioxidant, neuroprotective, hepatoprotective, and antimicrobial activities [25]. Additionally, gallic acid hexoside (**1**) shows potential as a treatment for cataracts deriving from diabetes mellitus [26]. Other compounds such as the monoterpene lactone loliolide (**53**), detected in the stems and leaves of *A. pyrifolia*, have been reported to possess anti-inflammatory properties [27], along with antioxidant, antifungal, antibacterial [28], and anticancer properties [29].

Moreover, flavonoids with pyrogallol or catechol B-ring moieties are known for their inhibitory activities against insect phenoloxidase and mushroom tyrosinase, crucial in plant defense against oxidative stress [18]. Also, flavonoids, as well as phenylpropanoid amides isolated from *A. frutescens*, exhibit significant radical scavenging activity compared to the reference trolox [18]. According to the authors, the presence of pyrogallol B-ring and *O*-acetyl at C-8 enhances the radical scavenging activity of the isolated molecules. These characteristics are found in the major compounds of *A. pyrifolia* and may contribute to the increased percentage of inhibition of the leaf’s extracts in both antioxidant assays.

Flavonoids are widely researched for their protective effects against oxidative stress. The antioxidant capacity of flavonoids is linked to their molecular structure, specifically the number and position of hydroxyl groups, conjugation and resonance effects, the surrounding environment that influences the preferred antioxidant site, and the unique antioxidant mechanism of each compound [30]. Their antioxidant properties have been associated with the hydrogen-donating ability of the B-ring catechol group and other structural features, including 2,3-unsaturation conjugated with a 4-oxo group in the C-ring [31]. Some of the characteristics of the major compounds have been identified in *A. pyrifolia*.

Several studies have demonstrated the non-toxicity of plant extracts rich in flavonoids against *A. salina* [32,33]. These flavonoids, known for their antioxidant properties, contribute to the protective effects observed in these bioassays. Most clinical reports suggest that flavonoid consumption is generally safe [30]. However, the increasing use of supplements raises concerns about potential toxicity and interactions with other substances or medications, which could lead to adverse effects [30]. The non-toxicity of plant extracts, as demonstrated through *Artemia salina* bioassays, suggests a favorable safety profile, which is an important preliminary indicator of safety for potential therapeutic applications in humans.

*A. salina* bioassays are widely recognized in general toxicity assessment [34]. Notably, *Artemia* shares genetic similarities with humans, particularly in the conserved Heat Shock Protein 70 (Hsp70) family, which plays a key role in stress responses to environmental toxins [35]. The similarity of Hsp70 genes between *Artemia* and humans suggests *Artemia* could serve as a valuable model. Studies have shown good correlation between *A. salina* lethality and in vitro cytotoxicity, highlighting its potential for the initial toxicological analysis of new compounds [36]. However, as human toxicity involves factors not fully mirrored in invertebrate models, these assays are preliminary. Further in vitro and in vivo mammalian studies are essential to confirm the safety and efficacy of these extracts for human use. This finding underscores the significance of initial safety assessments for plant-based extracts in developing new pharmacological treatments, especially those with potential for diverse biological applications.

## 4. Materials and Methods

### 4.1. General Experimental Procedures

The phytochemical screening was performed on a liquid chromatographic system Agilent Series 1290 (Agilent Santa Clara, CA, USA), and the separation was achieved on an Poroshell EC-C18 column (2.1 × 150 mm, 2.7 µm-Agilent Santa Clara, CA, USA). The mass spectrometric analysis was performed with a QToF-MS-MS (Model #G6545B, Agilent Technologies, Santa Clara, CA, USA) equipped with an ESI source with Jet Stream technology. All the operations, acquisition and analysis of data were controlled using Agilent MassHunter Acquisition Software Ver. A.10.1 and processed with MassHunter Qualitative Analysis Software Ver. B.07.00. Acetonitrile, methanol, formic acid used are of HPLC certified grade with 99.9% of purity were purchased from Fisher Scientific (Thermo Fishcer Scientific, Fair Lawn, NJ, USA). Water was purified using a Milli-Q system (Millipore, Bedford, MA, USA).

### 4.2. Plant Material and Extraction

Aerial parts of the *A. pyrifolia* plant were collected from the Karakus Mountains in the Turkestan region of Kazakhstan (42°31′45.00′′ N, 70°06′20.0′′ E) in May of 2022. Identification and authentication of the plant was performed by Dr. Mikhail Danilov, from the Institute of Botany and Phytointroduction (Almaty, Kazakhstan). The collected plant was stored in the Al-Farabi KazNU, Almaty, Kazakhstan with voucher numbers 01-05/518. Stems were separated from the leaves, and the plant materials were air-dried at room temperature (25 ± 5 °C) and 60 ± 5% relative humidity. The drying process was conducted in the shade, using special frames, for 21 days. During this period, the leaves and stems were turned every two days to ensure uniform drying. The collected raw materials were checked for the content of such impurities as solid particles of soil, dirt, dust, and insects. All procedures were carried out in the Pharmacognosy Laboratory of Al-Farabi Kazakh National University.

For the biological assays, the methanolic extracts were obtained from the ground dried leaves and stems of *A. pyrifolia* andsubjected to ultrasonic extraction using a Bransonic 5800 series ultrasonic cleaner (Branson, Brookfield, CT, USA), as described previously [10].

### 4.3. LC-MS Analysis

#### 4.3.1. Sample Preparation

About 1 g of dried samples of *A. pyrifolia* stem and leaf, finely powdered, were weighed and subjected to ultra-sonication for 30 min using 4 mL methanol solvent individually. Further, these samples were centrifuged at 4700 rpm for 15 min. The supernatant solution was removed from stem and leaf samples and transferred into a volumetric flask of 10 mL. The procedure was repeated twice using 3 mL methanol each time. After a third time, the sample was extracted followed by centrifugation; the volume of the supernatant solution in the volumetric flask was adjusted to 10 mL using methanol diluent. Further, these sample solutions were filtered using 0.45 µ PTFE filters before beingsubjected to LC-QToF-MS analysis.

#### 4.3.2. Instrumentation and Analytical Conditions

The liquid chromatographic separation was achieved on an Poroshell EC-C18 column (2.1 × 150 mm, 2.7 µm), with a mobile phase of water with 0.1% formic acid (A) and acetonitrile with 0.1% formic acid (B) at a flow rate of 0.23 mL/min. The analysis was performed using the following gradient elution: 5→30% of B in 25 min; 30→50% of B up to 3 in; 50→80% of B in 35 min; reaching 100% of B in 38 min, maintaining up to 41 min; and going to an equilibration period of 5 min with 5% of B. The column temperature was 45 °C, with an injection volume of 1 µL.

The mass spectrometric analysis was performed with a QToF-MS-MS equipped with an ESI source with Jet Stream technology using the following parameters: drying gas (N2) flow rate, 13 L/min; drying gas temperature, 325 °C; nebulizer pressure, 20 psi, sheath gas temperature, 300 °C; sheath gas flow, 12 L/min; capillary voltage, 3000 V; nozzle voltage, 0 V; skimmer, 45 V; Oct RF V, 750 V; and fragmentor voltage, 150 V. All the operations, acquisition, and analysis of data were controlled using Agilent MassHunter Acquisition Software (Ver. A.10.1) and processed with MassHunter Qualitative Analysis Software (Ver. B.07.00). Each sample was analyzed in positive and negative modes over the range of *m*/*z* 50–1700 and an extended dynamic range. Accurate mass measurements were obtained through employing ion correction techniques using reference masses at *m*/*z* 121.0509 (protonated purine) and 922.0098 (protonated hexakis [1H, 1H, 3H-tetrafluoropropoxy] phosphazine or HP-921) in positive ion mode, while *m*/*z* 112.9856 (deprotonated trifluoroacetic acid-TFA) and 1033.9881 (TFA adducted HP-921) were used in negative ion mode. Samples were analyzed in all-ion MS-MS modes, where experiment 1 was carried out with the collision energy of zero and experiment 2 with a fixed collision energy of 45 eV.

### 4.4. Antioxidant Capacity Assesment

#### 4.4.1. DPPH Free Radical Scavenging Activity

Şahin et al.’s [37] method was used to calculate the DPPH radical scavenging effects of the samples. The samples were mixed with 1 mM DPPH solution in methanol. Trolox was used as a standard antioxidant agent. The samples were incubated in the dark for 30 min at room temperature, then their absorbance was measured at 515 nm. Each experiment was performed triplicate. Inhibition % calculations were made using the equation below:

% Inhibition = [A blank − A sample/A blank] × 100

#### 4.4.2. ABTS Free Radical Scavenging Activity

The Singleton et al. [38] method was used in the calculation of ABTS radical scavenging effects of the samples. Trolox was used as a standard substance. ABTS was produced by reacting 0.746 mM ABTS with 0.245 mM potassium persulfate solution and allowing the mixture to stand in dark at room temperature for 24–48 h before use. Samples were mixed with diluted ABTS solution (1:10) and ethanol; absorbance was measured at 73 m after 6 min. Inhibition % values were calculated using the equation below:

% Inhibition = [A blank − A sample/A blank] × 100

### 4.5. Cytotoxic Assay on A. salina

Cytotoxic activity of the methanolic plant extract of *A. pyrifolia* was studied using the survival method of *A. salina* crustaceans. The flask was filled with artificial sea water and A. salina eggs were added. The mixture was kept for 3 days with a gentle supply of air until the crustaceans hatched from the eggs. This was conducted at a temperature of 20 ± 5 °C, with a natural photoperiod. The salinity of the control artificial water was set at 8.0–8.5 (pH). Dilutions of the methanolic plant were made to reach concentrations of 10, 5 and 1 mg/mL. Larvae of 1 day old were used for the test, with a density of 20–40 individual per test tube. The larvae were exposed to the three different concentrations of the plant extract on individual assays. Actinomycin D was used as a comparison drug. The mortality percentage was determined in relation to the number of larvae killed using the plant extract and the number of total larvae.

### 4.6. Statistical Analysis

The results of the cytotoxicity assay were expressed as mean ± standard deviation (SD; *n* = 3). Significant differences (*p <* 0.05) between the analyzed plant extracts based on cytotoxic activity were determined using one-way analysis (ANOVA) followed by Tukey’s multiple comparison test. Correlation between the studied parameters was calculated using Pearson’s correlation test. Statistical analysis was performed using Statistica 13.1 software (StatSoft, Inc., Tulsa, OK, USA).

## 5. Conclusions

The prevalence of flavonoids, such as gallocatechin/epigallocatechin and feruloyldopamine, in the stems extract, alongside the identification of major compounds like 8-Acetoxy-3′,4′,5,5′-tetrahydroxy-7-methoxy-3-α-L-rhamnopyranosyloxyflavone, pyrifolin, and dehydroxypyrifolin in the leaves through LC-MS analysis, highlights the chemical diversity of *A. pyrifolia*, expanding our understanding of its phytochemical profile and contributing to the characterization and differentiation of this species from others within the *Atraphaxis* genus.

Moreover, the presence of various flavonoids and phenolic compounds underscores both the chemical consistency and variability within the genus, as well as potential pharmacological activities. The non-toxicity of these flavonoid-rich extracts was further confirmed through bioassays with *A. salina*, as no significant mortality was observed even at higher concentrations. These findings represent the initial steps in demonstrating the safety and potential efficacy of this medicinal plant, supporting its possible application in pharmaceuticals and nutraceuticals.

These results represent significantly advances in our understanding of the chemotaxonomy and pharmacology within the *Atraphaxis* genus, initiating future investigations into the therapeutic potential of these species.

## Figures and Tables

**Table 1:** LC-QToF-MS data for compounds from various extracts of *A. pyrifolia*.

#	RT (Min)	Compound Name ^Ref^	Mol. Formula	Exact Mass [M]	[M + H]^+^	Fragment Ions (+ve Mode)	[M-H]^−^	Fragment Ions (−ve Mode)	Parts	
									Stem	Leaf
Phenolic Compounds										
1	4.22	Gallic acid hexoside	C_13_H_16_O_10_	332.0743	-	-	331.0673 (331.0671) *	169.0144 [M-H-C_6_H_10_O_5_]^−^, 151.0035 [M-H-C_6_H_10_O_5_-H_2_O]^−^, 125.0244 [M-H-C_6_H_10_O_5_-CO_2_]^−^	+	+
2	4.55	Gallic acid	C_7_H_6_O_5_	170.0215	-	-	169.0141 (169.0142)	125.0242 [M-H-CO_2_]^−^	+	+
3	7.09	Vanillic acid hexoside	C_14_H_18_O_9_	330.0951	353.0842 (353.0843) *	169.0495 [M + H-Glc]^+^, 151.0390 [M + H-Glc-H_2_O]^+^	329.0873 (329.0878)	167.0350 [M-H-Glc]^−^, 153.0196 [M-H-Glc-CH_2_]^−^	+	+
4	8.32	Chlorogenic acid isomers	C_16_H_18_O_9_	354.0951	355.1026 (355.1024)	163.0391 [Caffeic acid+H-H_2_O]^+^	353.0872 (353.0878)	191.0552 [Quinic acid-H]^−^, 179.0354 [Caffeic acid-H]^−^, 173.0456 [Quinic acid-H-H_2_O]^−^, 135.0443 [Caffeic acid-H-CO_2_]^−^	+	+
5	10.68									
6	11.22									
7	12.73									
8	8.21	Glucosyringic acid	C_15_H_20_O_10_	360.1056	-	-	359.0989 (359.0984)	197.0451 [M-H-Glc]^−^, 123.0086	+	+
9	8.80	Coumaric acid hexoside	C_15_H_18_O_8_	326.1002	349.0987 (349.0894) [M + Na]^+^	165.0546 [M + H-Glc]^+^, 147.0446 [M + H-Glc-H_2_O]^+^	325.0932 (325.0929)	163.0401 [M-H-Glc]^−^, 119.0502 [M-H-Glc-CO_2_]^−^	+	+
10	11.00									
11	10.03	Coumaroylquinic acid	C_16_H_18_O_8_	338.1002	339.1062 (339.1074)	147.0428 [Coumaric acid + H-H_2_O]^+^, 119.0478 [Coumaric acid+H-H_2_O-CO]^+^	337.0937 (337.0929)	191.0565 [Quinic acid-H]^−^, 163.0404 [Coumaric acid-H]^−^, 119.0506 [Coumaric acid-H-CO_2_]^−^	+	+
12	10.20	Methyl gallate	C_8_H_8_O_5_	184.0372	185.0434 (185.0444)	-	183.0299 (183.0299)	124.0167 [M-H-CH_3_-CO_2_]^−^	+	+
Flavonoids										
13	9.45	Procyanidin B1/B2/B3/B4	C_30_H_26_O_12_	578.1424	579.1495 (579.1497)	409.0915, 287.0514, 163.0390, 127.0390	577.1356 (577.1351)	451.1044, 425.0886, 407.0784, 339.0884, 289.0725, 245.0826, 161.0250, 125.0251	+	+
14	9.90									
15	11.41									
16	11.85									
17	14.47	Galloyl-procyanidin B1/B2	C_37_H_30_O_16_	730.1534	731.1607 (731.1607)	579.1498, 395.0953, 289.0703	729.1463 (729.1461)	577.1341, 407.0776, 371.0987, 289.0717, 169.0140, 151.0395, 125.0245	+	+
18	6.93	Gallocatechin/ Epigallocatechin	C_15_H_14_O_7_	306.0740	307.0805 (307.0812)	289.0709, 139.0382	305.0664 (305.0667)	137.0238, 125.0238	+	+
19	9.73									
20	10.40	Catechin/Epicatechin	C_15_H_14_O_6_	290.0790	291.0856 (290.0863)	147.0431, 139.0381, 123.0430	289.0715 (289.0718)	159.0451, 137.0247, 123.0456, 109.0299	+	+
21	12.89									
22	12.47	Fisetinidol hexoside	C_21_H_24_O_10_	436.1369	-	-	435.1303 (435.1297)	273.0771, 149.0246, 123.0456, 109.0297	+	+
23	13.07	Quercetin 3-glucuronide-methyl ester	C_22_H_20_O_13_	492.0904	-	-	491.0836 (491.0831)	337.0933, 300.9996, 191.0563	ND	+
24	13.59	Heptahydroxyflavone; 7-Me ether, rhamnopyranoside, hexoside	C_28_H_32_O_18_	656.1589	657.1665 (657.1661)	511.1082, 495.1137, 349.0556, 209.1534, 183.0287	655.1534 (655.1518)	493.0985, 346.0334, 331.0097, 191.0201	+	+
25	14.00	Fisetinidol	C_15_H_14_O_5_	274.0841	275.0908 (275.0914)	123.0434	273.0776 (273.0768)	149.0247	+	+
26	15.00	Pentahydroxy-7-methoxyflavone; rhamnopyranoside, hexoside	C_28_H_32_O_17_	640.1639	641.1716 (641.1712)	495.1133, 479.1184, 155.1311, 434.2022, 333.0609	639.1589 (639.1567)	477.1058, 439.1086, 331.0467, 330.0394, 315.0156, 191.0569	+	+
27	15.30	Myricetin hexoside	C_21_H_20_O_13_	480.0904	481.0969 (481.0977)	319.0436, 153.018	479.0838 (479.0831)	316.0230, 271.0257	+	+
28	15.34	Guibourtinidol-(Cat)Epicatechin-2/3/4/5/6/7	C_30_H_26_O_10_	546.1526	547.1600 (547.1599)	271.0601, 147.0437, 123.0437	545.1463 (545.1453)	289.0726, 245.0824	+	+
29	16.75									
30	17.60									
31	17.90									
32	16.09	Trihydroxy-7-methoxyflavone; [Glucofuranosyl hexoside]	C_28_H_32_O_16_	624.1690	625.1771 (625.1763)	479.1186, 317.0658	623.1630 (623.1618)	477.1041, 461.1060, 315.0502, 314.0430, 299.0197, 181.0136	+	+
33	16.90	Heptahydroxyflavone; 7-Me ether, rhamnopyranoside/Pentahydroxy-methoxy-rhamnopyranosyloxy-flavone	C_22_H_22_O_13_	494.1060	495.1128 (495.1133)	349.0552, 334.0303, 317.0281, 303.0484, 235.0223, 183.0277, 169.0120, 153.0170	493.1008 (493.0998)	346.0341, 331.0109, 303.0157, 231.0303	+	+
34	17.32	Quercetin 3-*O*-glucuronide	C_21_H_18_O_13_	478.0747	479.0819 (479.0820)	303.0495, 137.0593	477.0684 (477.0675)	301.0358, 151.0037	+	+
35	17.40	Quercetin hexoside	C_21_H_20_O_12_	464.0955	465.1029 (465.1028)	303.0502, 153.0181	463.0890 (463.0882)	300.0282, 271.0253, 255.0302, 151.0035	+	+
36	17.00	Cassiaflavan-(Cat) Epicatechin-1/2/3/4	C_30_H_26_O_9_	530.1577	531.1648 (531.1650)	271.0596	529.1518 (529.1504)	289.0723	+	+
37	17.95									
38	18.70									
39	19.75									
40	19.00	Pyrifolinin	C_22_H_22_O_12_	478.1111	479.1186 (479.1184)	353.0263, 333.0600, 318.0363, 301.0338, 169.0124, 137.0227	477.1052 (477.1038)	331.0462, 315.0154, 287.0202, 271.0254, 181.0146	+	+
41	19.30	Kaempferol hexoside	C_21_H_20_O_11_	448.1006	449.1078 (449.1078)	287.0550	447.0931 (447.0933)	285.0392, 284.0324, 255.0301, 227.0353	+	+
42	20.96	3,4′,5,7,8-Pentahydroxyflavone; 7-Me ether, 3-*O*-α-L-rhamnopyranoside	C_22_H_22_O_11_	462.1162	463.1242 (463.1235)	355.0132, 337.0324, 317.0658, 302.0424	461.1097 (461.1089)	315.0511, 299.0204, 271.0254, 255.0308, 133.0298	ND	+
43	23.59	3,3′,4′,5,5′,7,8-Heptahydroxyflavone; 7-Me ether, 8-Ac, 3-*O*-α-L-rhamno-pyranoside/8-Acetoxy-3′,4′,5,5′-tetrahydroxy-7-methoxy-3-α-L-rhamno-pyranosyloxyflavone	C_24_H_24_O_14_	536.1166	537.1249 (537.1239)	391.0704, 349.0556, 334.0328, 317.0298, 183.0292, 153.0186, 139.0394	535.1105 (535.1093)	493.0995 [M-H-C_2_H_2_O]^−^, 388.0442 [M-H-Rha]^−^, 373.0204 [M-H-Rha-CH_3_]^−^, 331.0115 [M-H- C_2_H_2_O -Rha-CH_3_]^−^, 303.0145 [M-H- C_2_H_2_O -Rha-CH_3_-CO]^−^, 181.0139 [C_8_H_6_O_5_-H]-	+	+++
44	24.28	Pyrifolinin	C_22_H_22_O_12_	478.1111	479.1181 (479.1184)	330.0385, 315.0148, 287.0202, 165.0194, 137.0237	477.1043 (477.1038)	333.0601, 259.0595, 231.0644, 167.0331, 139.0383	ND	+
45	26.26	Pyrifolin	C_24_H_24_O_13_	520.1217	521.1290 (521.1291)	397.0520, 375.0696, 333.0598, 318.0361, 301.0329, 273.0371, 183.0271, 169.0114, 137.0216	519.1151 (519.1144)	Ester Bond breakage: 477.1043 [M-H-C_2_H_2_O]^−^, 373.0563 [M-H-Rha]^−^, 372.0488, 357.0250 [M-H-C_6_H_10_O_4_-CH_4_]^−^, 315.0152 [M-H-C_6_H_10_O_4_-CH_4_-CO_2_]^−^, 287.0192 [M-H-C_6_H_10_O_4_-CH_4_-CO_2_-CO]^−^, 271.0243 [M-H-C_6_H_10_O_4_-CH_4_-2CO_2_]^−^, 181.0137 [C_8_H_6_O_5_-H]^−^	+	+++
46	28.28	Dehydroxypyrifolin	C_24_H_24_O_12_	504.1268	505.1358 (505.1341)	381.0586, 359.0771, 317.0670, 302.0441, 285.0398, 183.0299,	503.1204 (503.1194)	357.0611, 341.0303, 299.0214, 271.0247, 255.0297, 181.0141	+	+++
Catecholamines										
47	16.36	Feruloyl-noradrenaline	C_18_H_19_NO_6_	345.1212	346.1287 (346.1285)	328.1188,177.0547, 145.0284, 117.0335	344.1145 (344.1140)	326.1039, 161.0244, 149.0488	+	+
48	18.01	Feruloyldopamine	C_18_H_19_NO_5_	329.1263	330.1333 (330.1336)	312.1226, 177.0543, 145.0281, 117.0333	328.1194 (328.1190)	161.0247, 133.0535	+++	+
49	20.66					177.0541, 145.0280	328.1194 (328.1190)	-	+	+
50	19.60	Coumaroyldopamine	C_17_H_17_NO_4_	299.1158	300.1231 (300.1230)	-	298.1087 (298.1085)	-	+	+
51	22.25	Paprazine	C_17_H_17_NO_3_	283.1208	284.1264 (264.1281)	177.0439, 147.0337, 121.0549	282.1146 (282.1136)	-	+	+
52	23.30	Feruloyltyramine	C_18_H_19_NO_4_	313.1314	314.1388 (314.1387)	177.0546, 145.0280, 117.0335	312.1246 (312.1241)	-	+	+
Monoterpenoid lactone										
53	17.80	Loliolide	C_11_H_16_O_3_	196.1099	197.1172 (197.1172)	179.1066, 105.0697	-	-	+	+

* Theoretical accurate mass; ‘+’ indicates presence of compound; ‘ND’ indicates not detected; ‘+++’ indicates strong intensity.

**Table 2:** Antioxidant capacity of *A. pyrifolia* leaves and stems methanolic extract.

Extract	DPPH (mg Trolox/g)	ABTS (mg Trolox/g)
Leaves	403.85 ± 18.49	124.22 ± 3.24
Stems	365.69 ± 0.68	14.45 ± 2.98

**Table 3:** Results of the cytotoxic activity study against *A. salina*.

Treatments	Concentrations (mg/mL)	% of Surviving Larvae	Mortality (%)
Negative control	-	100	0
	-	100	0
	-	100	0
Actinomycin D	10	0	96 *
	5	4	92 *
	1	33	63 *
Methanolic leaves extract	10	96	0
	5	96	0
	1	96	0

* *p <* 0.05 compared to negative control.

## Data Availability

Data are contained within the article.
